# Supplementary material for: Obesity-associated NLRC4 inflammasome activation drives breast cancer progression
Source: Nat Commun. 2016 Oct 6;7:13007. doi: 10.1038/ncomms13007 (PMC5059727; doi:10.1038/ncomms13007)
Supplement: Supplementary Information — Supplementary Figures 1-7 and Supplementary Table 1 [file ncomms13007-s1.pdf]

SUPPLEMENTARY FIGURES

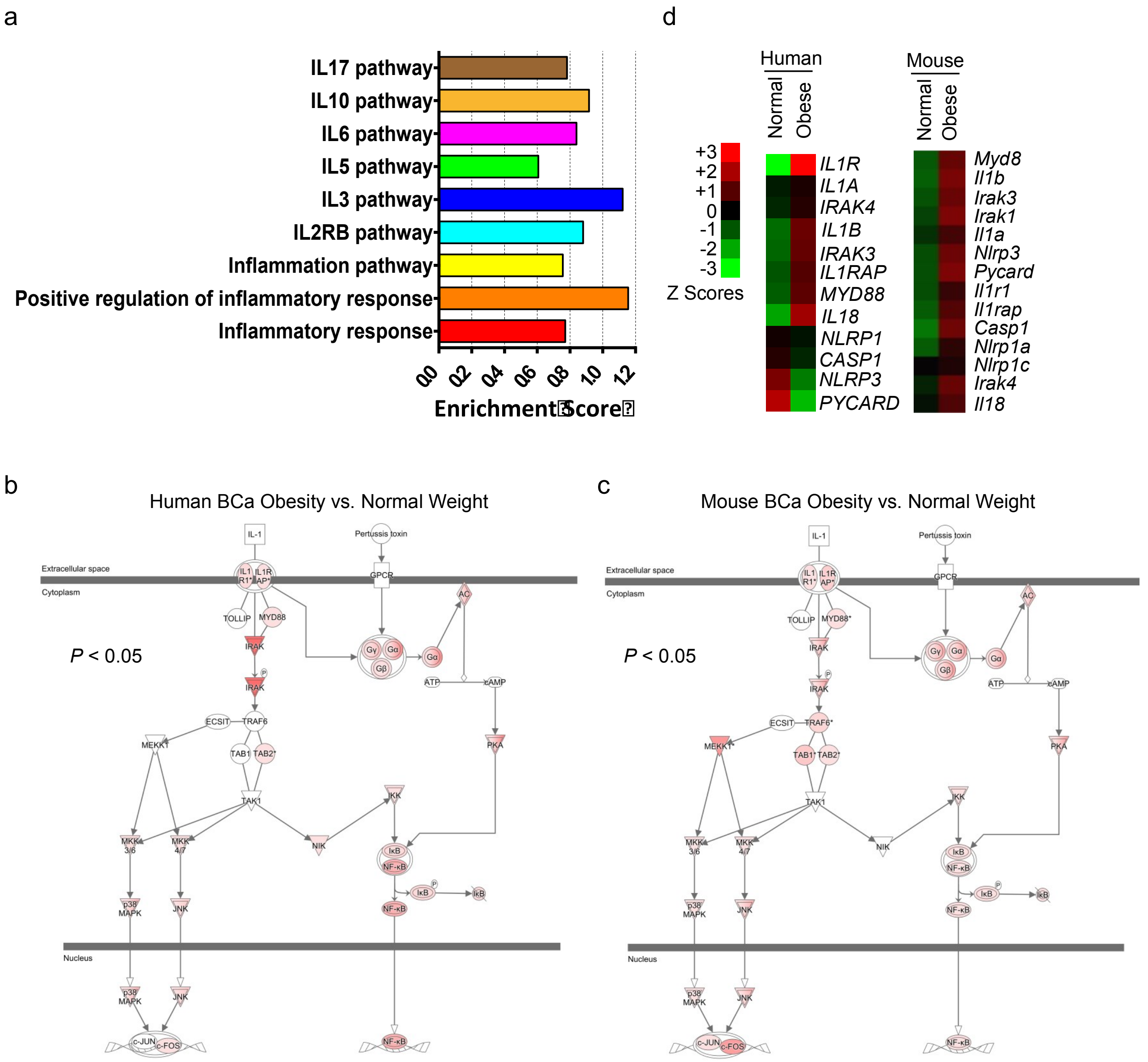

Supplementary Figure 1. The pro-inflammatory IL-1/IL-1R1 signaling pathway is elevated in breast-cancer specimens from obese patients and animals. (a) Transcriptomic analysis revealed significant upregulation of inflammatory pathways in the tumors of ER+ obese breast cancer patients (n=43) in comparison to those of ER+ non-obese breast cancer patients (n=94) (GSE20194, Gene Expression Omnibus). Data represents the enrichment score of the indicated pathway. **(b-c)** Transcriptomic analysis revealed significant upregulation of genes involved in IL-1 signaling in the tumors of ER+ obese breast cancer patients (n=43) in comparison to those of ER+ non-obese breast cancer patients (n=94) (GSE20194, Gene Expression Omnibus) (**b**), or in the mammary tumors extracted from MMTV-*TGFα; Ay/a* obese mice (n=5) compared to those of MMTV-*TGFα*; *a/a* lean mice (n=4) (**c**). (**b-c**) Red color indicates the upregulation of indicated genes. (**d**) Z-scores of the mean gene expression involved in IL-1 signaling from cancer specimens of obese patients or obese mice relative to those from normal weight controls. A heat map of the Log_2 Z-score for the genes is included with green-black-red color schemes. Significance was determined by right-tailed Fisher exact test. n=43 for obese human specimens and n=94 for non-obese human specimens. n=5 for obese mouse tumors and n=4 for non-obese mouse tumors.

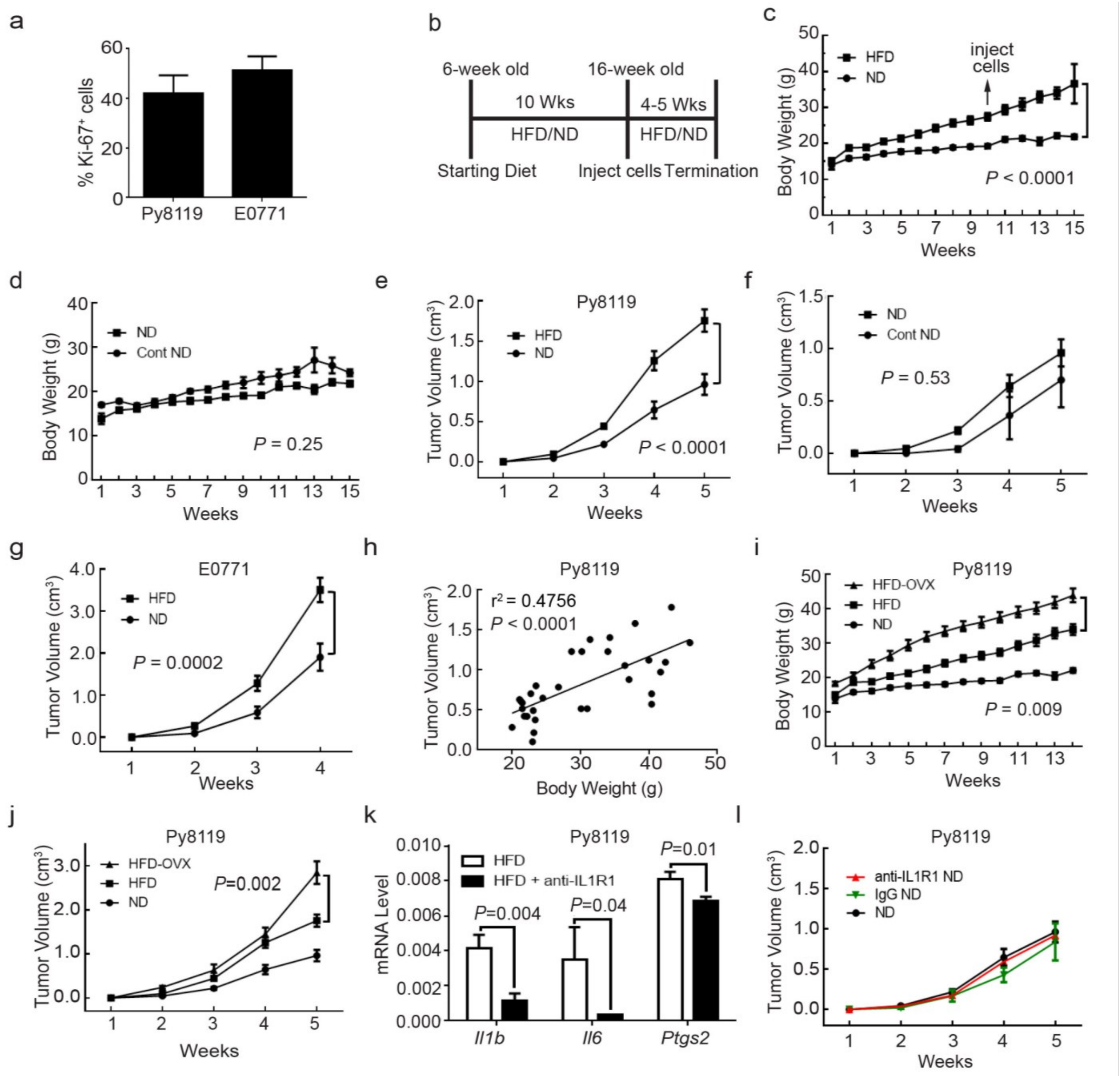

Supplementary Figure 2. Obesity drives breast-cancer growth *in vivo*. (a) Data represents the % of Ki-67-positive cells from Py8119 and E0771 derived tumors ± s.d. **(b)** Experimental outline. Six-week C57BL/6 mice are fed a nutrient control ND, ND or HFD for 10 weeks. Py8119 and E0771 cells are transplanted into the mammary gland. Body weight and tumor volume are monitored weekly. **(c-g)** Mice were treated as in **(b)**. Data represents the mean body weight **(c-d)** and tumor volume **(e-g)** ± s.e.m. of the indicated mice. (n=5 Cont ND; n= 14 ND; n=24 HFD; E0771: n=5 ND; n=5 HFD). Two-way Anova was used to determine significance. **(h)** Linear regression of body weight versus tumor volume for Py8119 derived tumors, including both ND- and HFD-fed WT female mice from **(e)**. Pearson's coefficient is indicated and significance was determined by F test. (**i-j)** C57BL/6 mice or ovariectomized C57BL/6 mice (OVX) were treated as in **(b)**. Data represents the mean body weight **(i)** and Py8119 tumor volume **(j)** ± s.e.m. (n=14 ND; n=24 HFD; n=6 HFD-OVX). Two-way Anova was used to determine significance. **(k)** Data represents the average expression (relative to *Ppia*) of the indicated genes ± s.d. from the indicated tumors (n=3 for all groups). Group means were compared by Student's t test to determine significance. **(l)** Mean tumor volume ± s.e.m. from the indicated mice fed with ND.

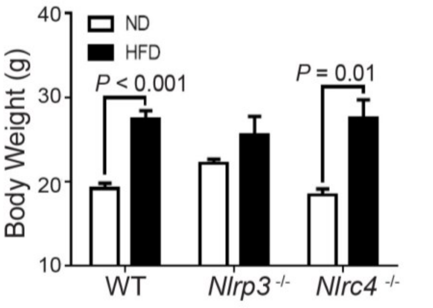

Supplementary Figure 3. NLRC4-deficiency has no impact on HFD-induced weight gain. Mean body weight ± s.e.m. from the indicated mice after 10 weeks of HFD or ND (WT: n=10 ND, n=14 HFD; *Nlrp3*$^{-/-}$: n=5 ND, n=4 HFD; *Nlrc4*$^{-/-}$: n=5 ND, n=9 HFD). Group means were compared by Student's t test to determine significance.

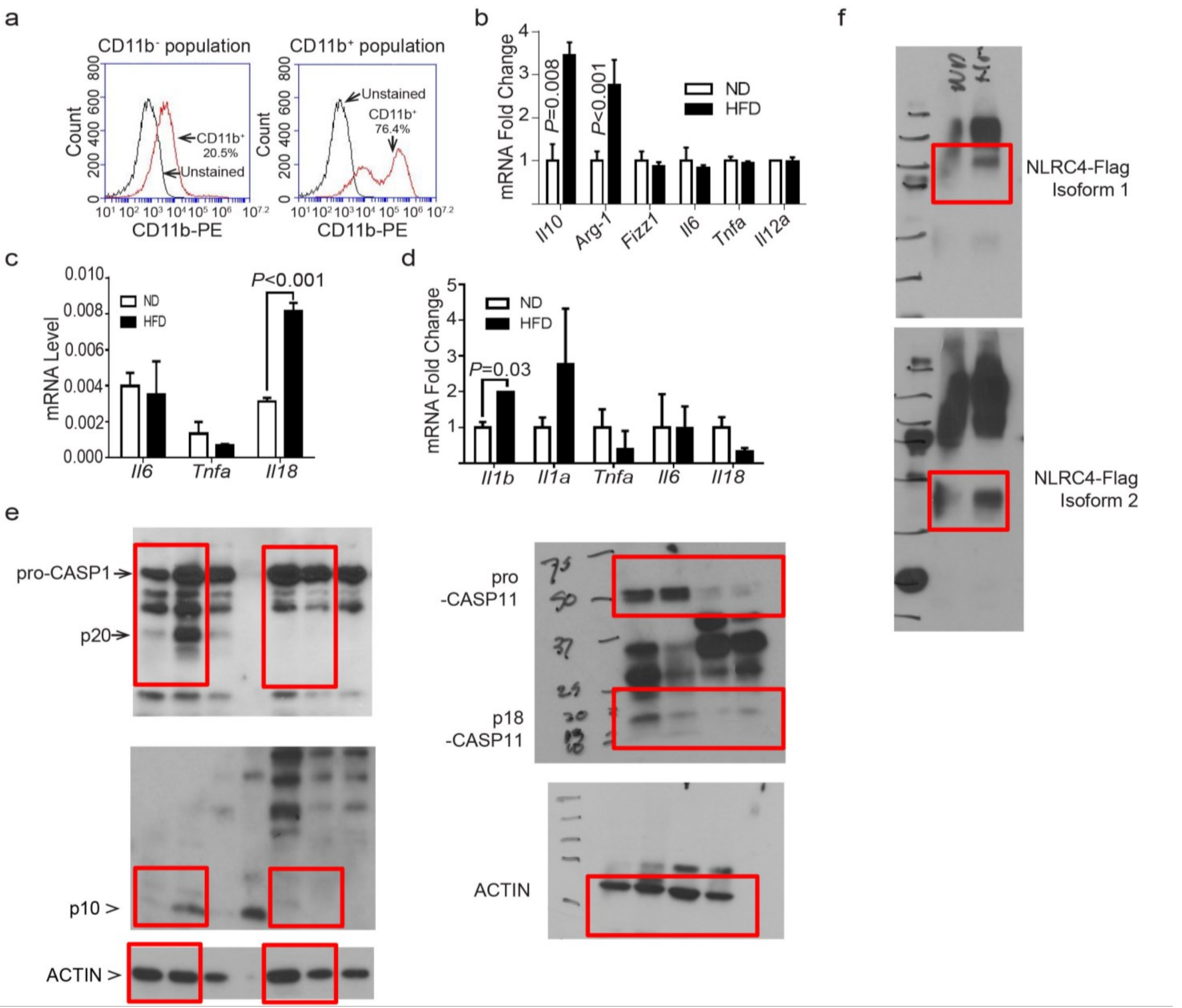

Supplementary Figure 4. Obesity induces NLRC4 inflammasome in tumor-infiltrating myeloid cells. (a) Histograms from a positive selection for CD11b$^+$ cells from E0771 tumors. The percentages of CD11b$^+$ cells from the negative and positive populations are indicated. **(b)** Data represents the mRNA fold change compared to the ND group of the indicated genes ± s.d. in CD11b$^+$ cells **(a)** from ND and HFD mice. Group means were compared by Student's t test to determine significance (n=3 for all groups). **(c-d)** mRNA expression (relative to *Ppia*) of the indicated genes ± s.d. in Py8119 **(c)** or E0771 **(d)** tumors from the indicated mice (n=3 for all groups). Group means were compared by Student's t test to determine significance. **(e)** Supplementary data for full gel images for Fig. 3c. Western blot analysis for CASP1 (left panels) and CASP11 (right panels) in tumor-infiltrating CD11b$^+$ and CD11b$^-$ cell populations. Cells were combined from 4-5 tumors from each group. **(f)** Supplementary data for full gel images for Fig. 3g. Western blots for NLRC4-flag in tumor infiltrating CD11b$^-$ and CD11b$^+$ cell populations from DIO mice.

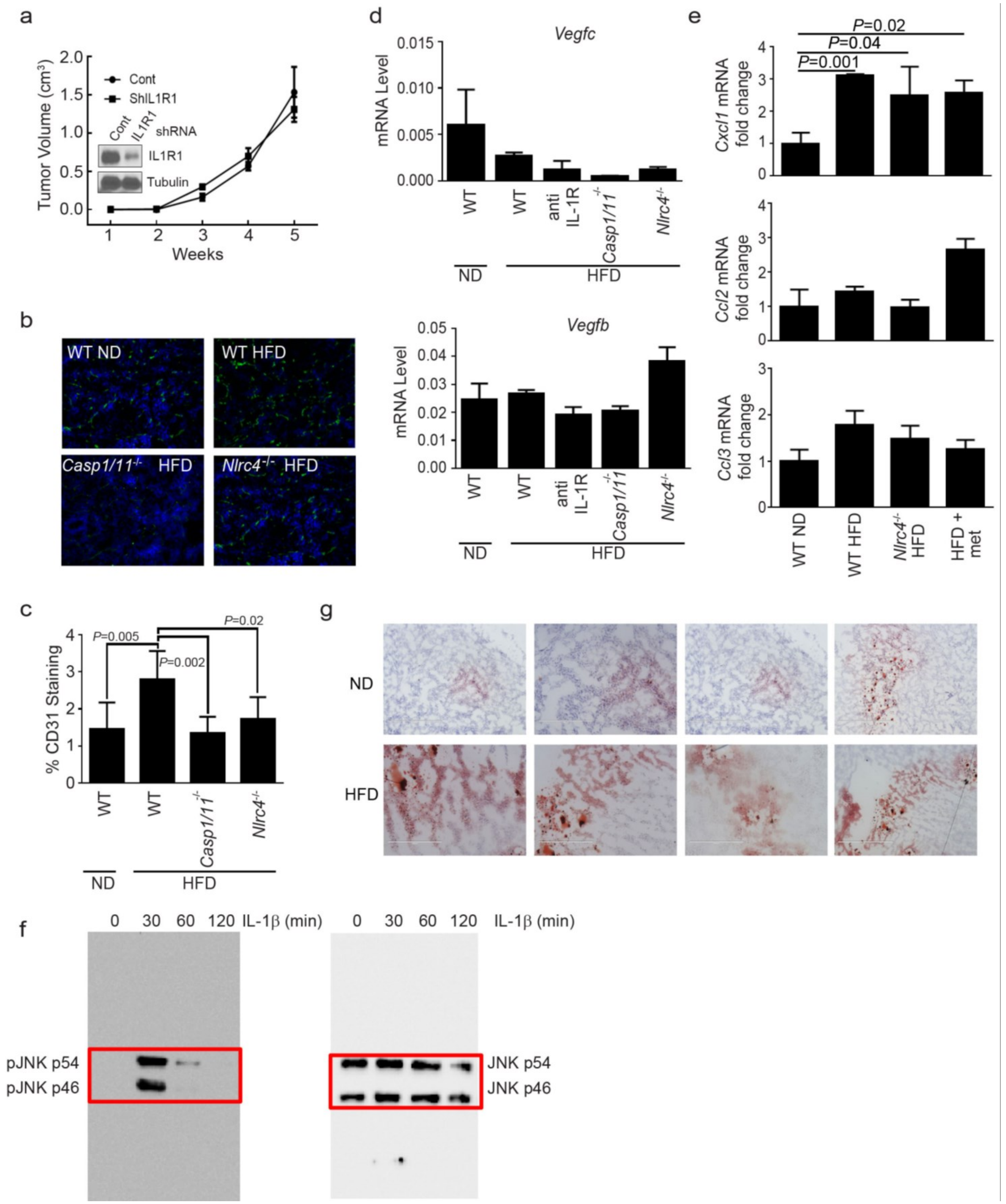

Supplementary Figure 5. NLRC4 inflammasome promotes angiogenesis in diet-induced obese mice. (a) Py8119 cells were infected with lentiviral particles encoding IL1R1 specific shRNA or control shRNA. Cells were stably selected and then injected in to the mammary gland of mice fed a HFD for 10 weeks. Inlet: western blot analysis showing decreased IL1R1 expression by shRNA. Data represents the mean tumor volume ± s.e.m (n=5 all groups). **(b)** Representative picture of immunofluorescent (IF) staining for CD31 in Py8119 tumors

from the indicated mice. **(c)** Quantification of IF staining in **(b)**. Data represents the average area of CD31-positive staining over total area ± s.d. At least 3 fields per section and 3 tumors per group were used in the analysis. Group means were compared by Student's t test to determine significance. **(d)** Mean mRNA expression of *Vegfc* and *Vegfb* in Py8119 tumors from the indicated mice ± s.d. (n=3 for all groups). **(e)** Mean mRNA expression of *Cxcl1, Ccl2 and Ccl3* in tumors from the indicated mice ± s.d. (n=3 for all groups). **(f)** Supplementary data for full gel images for Fig. 5e. Primary mammary adipocytes were treated with 100ng/ml rIL-1β for the indicated time, and the indicated proteins were separated by SDS-PAGE followed by immunoblotting with the indicated antibodies. **(g)** Py8119 tumor sections from the ND or HFD groups were stained with Oil-O Red stain to identify lipid droplets in the tumor. Representative images from 4 different tumors within each group were shown. Scale bar: 200μm.

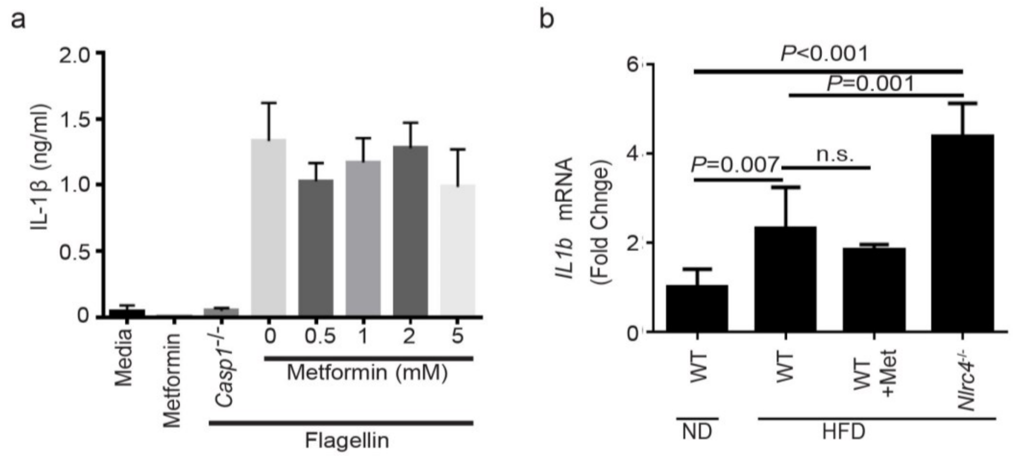

Supplementary Figure 6. Metformin inhibits tumor growth and angiogenesis in diet-induced obese mice. **(a)** BMDM from wild type (WT) or *Casp1/11*$^{-/-}$ mice were primed with LPS then treated with the indicated doses of metformin. The indicated cells were transfected with flagellin for 8 hr and IL-1β levels in the supernatant were determined by ELISA. Data represents the mean IL-1β concentration ± s.d. **(b)** Mean mRNA expression of *IL1b* (relative to *Ppia*) in Py8119 tumors from the indicated mice presented as fold change compared to WT ND ± s.d. (n=3 for all groups). Group means were compared by Student's t test to determine significance.

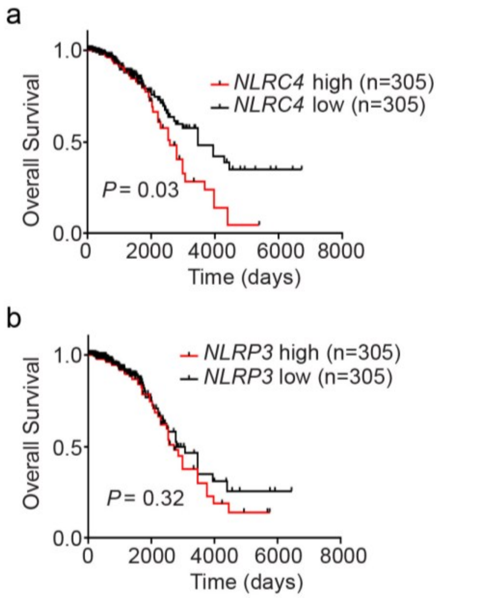

Supplementary Figure 7. NLRC4 expression but not NLRP3 is associated with shorter overall survival. (a-b) TCGA invasive ductal carcinoma dataset was downloaded and analyzed for the correlation *NLRC4* or *NLRP3* expression with overall survival in breast cancer patients. Kaplan-Meier survival curve was drawn via Graphpad Prism, only using the high versus low tertiles of *NLRC4* or *NLRP3* expression for comparison. Statistical significance was determined by Log-rank test with *P* values and n numbers indicated.

Supplementary Table 1: Primer list for real-time PCR.

Gene	Sequence
Actb	ATGGAGGGGAATACAGCCC
	TTCTTTGCAGCTCCTTCGTT
Arg1	TTTTTCCAGCAGACCAGCTT
	AGAGATTATCGGAGCGCCTT
Fizz1	AAGCCTACACTGTGTTTCCTTTT
	GCTTCCTTGATCCTTTGATCCAC
Il10	GGTTGCCAAGCCTTATCGGA
	ACCTGCTCCACTGCCTTGCT
Il12	GCTTCTCCCACAGGAGGTTT
	CTAGACAAGGGCATGCTGGT
Il18	GACTCTTGCGTCAACTTCAAGG
	CAGGCTGTCTTTTGTCAACGA
Il1a	CGAAGACTACGTTCTGCCATT
	GACGTTTCAGAGGTTCTCGAG
Il1b	GCAACTGTTCCTGAACTCAACT
	ATCTTTTGGGGTCCGTCAACT
Il1r1	GTGCTACTGGGGCTCATTTGT
	GGAGTAAGAGGACACTTGCGAAT
Il6	TAGTCCTTCCTACCCCAATTTCC
	TTGGTCCTTAGCCACTCCTTC
Nlrc4	ATCGTCATCACCGTGTGGAG
	GCCAGACTCGCCTTCAATCA
Nlrp3	ATTACCCGCCCGAGAAAGG
	TCGCAGCAAAGATCCACACAG
Ppia	CAGTGCTCAGAGCTCGAAAGT
	GTGTTCTTCGACATCACGGC
Ptgs2	GGCGCAGTTTATGTTGTCTG
	CAGCACTTCACCCATCAGTT
Tnfa	ATGAGAGGGAGGCCATTTG
	CAGCCTCTTCTCATTCCTGC
Vegfa	AATGCTTTCTCCGCTCTGAA
	GCTTCCTACAGCACAGCAGA
Vegfb	GTGAAGCAGGGCCATAAAAG
	GAGCTCAACCCAGACACCTG
